# Comparison between P-POSSUM and NELA risk score for patients undergoing emergency laparotomy in Egyptian patients

**DOI:** 10.1186/s12893-023-02189-y

**Published:** 2023-09-21

**Authors:** Mahmoud Magdy Alabbasy, Alaa Abd Elazim Elsisy, Adel Mahmoud, Saad Soliman Alhanafy

**Affiliations:** 1 Department of General Surgery, Faculty of Medicine, Menoufia University, Shebin-Elkom, Menoufia Egypt; 2 Laparoscopic Colorectal Surgery Fellow, Swansea Bay University Health Board, Swansea, UK

**Keywords:** Emergency laparotomy, The area under the curve, Mortality, Outcomes, Pairwise comparisons

## Abstract

**Background and aims:**

The Portsmouth-Physiological and Operative Severity Score for the enumeration of Mortality and Morbidity (P-POSSUM) is one of the scores that is used most frequently for determining the likelihood of mortality in patients undergoing emergency laparotomy. National Emergency Laparotomy Audit (NELA) presents a novel and validated score. Therefore, we aimed to compare the performance of the NELA and P-POSSUM mortality risk scores in predicting 30-day and 90-day mortality in patients undergoing emergency laparotomy.

**Methods:**

Between August 2020 and October 2022, this cohort study was undertaken at Menoufia University Hospital. We compared the P-POSSUM, preoperative NELA, and postoperative NELA scores in patients undergoing emergency laparotomy. All variables needed to calculate the used scores were collected. The outcomes included the death rates at 30 and 90 days. By calculating the area under the curve (AUC) for every mortality instrument, the discrimination of the various methods was evaluated and compared.

**Results:**

Data from 670 patients were included. The observed risk of 30-day and 90-day mortality was 10.3% (69/670) and 13.13% (88/670), respectively. Concerning 30-day mortality, the AUC was 0.774 for the preoperative NELA score, 0.763 for the preoperative P-POSSUM score, and 0.780 for the postoperative NELA score. Regarding 90-day mortality, the AUCs for the preoperative NELA score, preoperative P-POSSUM score, and postoperative NELA score were 0.649 (0.581–0.717), 0.782 (0.737–0.828), and 0.663 (0.608–0.718), respectively. There was noticeable difference in the three models' capacity for discrimination, according to pairwise comparisons.

**Conclusions:**

The probability of 30-day and 90-day death across the entire population was underestimated by the NELA and P-POSSUM scores. There was discernible difference in predictive performance between the two scores.

## Introduction

Emergency laparotomy results in approximately 10 times more fatalities than major elective gastrointestinal surgery, which is a frequent urgent surgical procedure [1]. Several mortality prediction models have been devised to show the risk of death if an emergency laparotomy had been performed. These tools have also been utilized to alter patient cohorts for risk to allow comparison of outcomes from different medical facilities. A good example of one of these models is a strategy for calculating mortality and morbidity based on the Portsmouth-physiological and operative severity (PPOSSUM) score [2, 3]. Because of its beginnings, the P-POSSUM prediction scoring system has become the most widely used and extensively verified tool in the field of perioperative care [4]. P-POSSUM, however, has several shortcomings. First, it has been revealed that P-POSSUM overestimates mortality [5, 6] and is inaccurate in several patient groups undergoing emergency laparotomy when used either preoperatively or postoperatively [7]. Second, comprehensive research [5, 7] has shown that the model's design has flaws. After realizing the limitations of the P-POSSUM score, the National Emergency Laparotomy Audit (NELA) group [8] developed a risk assessment model based on information from more than 38,000 cases in the UK NELA to forecast 30-day mortality in patients undergoing emergency laparotomy. It has been shown that the NELA model, which uses a combination of risk variables gathered through standard clinical procedures, has excellent calibration and discriminatory power [8]. The NELA risk model has, however, only been approved by Australia and the United Kingdom [8, 9]. Therefore, we aimed to compare the performance of the NELA mortality hazard score and the P-POSSUM score in predicting 30-day and 90-day mortality in Egyptian patients who underwent emergency laparotomy.

## Patients and methods

This prospective cohort study was conducted at the general surgery department, Menoufia University Hospital, Egypt. All adult patients aged over 18 who underwent emergency laparotomy between 1st August 2020 and 1st October 2022, were deemed eligible for this study. Patients under the age of 18, patients underwent laparotomies or laparoscopies on an elective basis and those who did not have all the required investigations to calculate the NELA or P-POSSUM scores were excluded. Patient data such as age, sex, comorbidities, clinical examination, laboratory investigations especially (complete blood count, serum albumin, serum urea, and serum electrolytes as Sodium and potassium), ECG, chest X-ray, American Society of Anesthesiology (ASA) score, Glasgow coma scale, clinical urgency, surgical indications and operative parameters needed to determine the NELA and P-POSSUM scores, as well as the 30- and 90-day mortality, were gathered and analyzed [10, 11]. Procedure-specific sub-analysis has not been conducted because both scores are general risk models designed to be used on a range of procedures [12]. We compared the outcomes for the subsequent pairwise comparisons: (a) Preoperative P-POSSUM score in comparison to preoperative NELA score. (b) A comparison of the NELA scores before and after surgery. (c) Comparing the postoperative NELA and preoperative P-POSSUM scores. The outcomes involved 30-day and 90-day after-surgery mortality, which was defined as death from any reason within 30 and 90 days, respectively, following an emergency laparotomy. The research was performed with the approval of the Research Ethics Committee, Faculty of Medicine, Menoufia University, Egypt; (IRB. 2/2023SURG28-3), and by the Helsinki Declaration. Moreover, informed consent was obtained from all patients included in this study before the surgical procedure.

### Sample size estimation

The sample size was calculated to include all patients who underwent emergent laparotomy, fulfilling all inclusion and exclusion criteria, and were collected from Menoufia University hospitals over two years. The least sample size calculated using statistics and sample size pro was 416 participants. The power of the study is 80% and the confidence interval is 95% (calculated by G power Program).

### Statistical analyses

The statistical analysis was performed with SPSS Statistics version 25 by IBM Corp. (Armonk, NY, USA). The categorical variables were summarized using absolute and relative frequencies and compared using the chi-square test. The continuous variables were summarized using the median and compared using the Monte Carlo test and Mann–Whitney U test. For every predicted model, we determined the observed deaths to expected deaths ratio (O/E ratio) by dividing the actual mortality rate (observed) by the calculated mortality score (expected). The O/E ratio would help assess whether the risk-prediction tool overestimates or underestimates the risk of postoperative mortality. Analyses using the receiver operating characteristic (ROC) curve were carried out to measure and contrast the mortality tools' discriminatory abilities by calculating the AUC for every mortality tool. Diagnostic Odds Ratio (DOR) and Bootstrap were used to estimate the uncertainty in AUC and other performance metrics. At the 5% level of significance, the results were found to be substantial.

## Results

A total of 724 patients underwent emergency laparotomy at our hospitals between August 2020 and October 2022. 54 patients of them were excluded because they did not have all the required investigations to calculate the NELA or P-POSSUM scores. Therefore, only 670 patients were included in our current study. Patients` age was from 18 to 91, and 45.7% (306/670) of them were male. The classification of the ASA score is shown in Table 1.

**Table 1:** Characteristics of the involved patients regarding 30-day and 90-day mortality

Variables		Total *n* = 670	Mortality in 30 days		Mortality in 90 days	
			**no *n* = 601**	**yes *n* = 69**	**no *n* = 582**	**yes *n* = 88**
**Age**	Mean ± SD	61.15 ± 17.503	60.04 ± 17.682	70.70 ± 12.349	59.80 ± 17.770	69.29 ± 13.201
	Mann–Whitney test value		14,939.500		20,167.5	
	*P* value		0.000		0.001	
**Gender**	Male	306 (45.67%)	275 (89.9%)	31 (10.1%)	264(86.3%)	42(13.7%)
	Female	364(54.33%)	326 (89.6%)	38 (10.4%)	318(87.4%)	46(12.6%)
	Pearson Chi-Square test value		0 .017		0 .173	
	*P* value		0 .896		0 .678	
	Odds Ratio DOR		1.034 (0.627–1.706)		0 .909(0.580–1.425)	
**ASA score**	I	65(9.7%)	65(100.0%)	0(0.0%)	65(100.0%)	0(0.0%)
	II	240(35.82%)	233(97.1%)	7(2.9%)	227(94.6%)	13(5.4%)
	III	237(35.37%)	217(91.6%)	20(8.4%)	213(89.9%)	24(10.1%)
	IV	113(16.86%)	78(69.0%)	35(31.0%)	71(62.8%)	42(37.2%)
	V	15(2.25%)	8(53.3%)	7(46.7%)	6(40.0%)	9(60.0%)
	Monte Carlo test		80.21		92.010	
	*P* value		0.000		0.000	
**ASA score Odds Ratio DOR 95.0% C.I**	I	65 (9.7%)	0.000 (0.000–0.000)		0.000 (0.000–0.000)	
	II	240 (35.82%)	0.034 (0.010–0.121)		0.038 (0.012–0.124)	
	III	237 (35.37%)	0.105 (0.035–0.321)		0.075(0.025–0.229)	
	IV	113 (16.86%)	0.513(0.172–1.525)		0.394 (0.131 -1.186)	
	V	15(2.25%)	**References**		**References**	
**Preoperative NELA**	Mean ± SD	34.81 ± 34.05	22.17 ± 29.18	40.26 ± 43.94	34.15 ± 33.03	40.14 ± 39.51
	Minimum–Maximum	(0.01–84.9)	(0.01–75.3)	(0.6–84.9)	(0.01–75.3)	(0.6–84.9)
	Median	3.2	2.7	17.2	2.6	15.4
	Mann–Whitney test value		9371.500		16,522.000	
	*P* value		0.000		0.000	
**Postoperative NELA**	Mean ± SD		23.04 ± 35.25	42.42 ± 46.33	43.91 ± 37.55	70.85 ± 19.92
	Minimum–Maximum	(0.01–98.7)	(0.01–98.7)	(0.12–98)	(0.01–98.7)	(0.12–98)
	Median	4.8	4.4	23.7	4.1	17.1
	Mann–Whitney test value		9139.000		17,234.000	
	*P* value		0.000		0.000	
**Preoperative P-POSSUM**	Mean ± SD		22.79 ± 34.74	34.74 ± 37.37	20.65 ± 32.68	45.29 ± 45.03
	Minimum–Maximum	(0.01–96.7)	(0.01–96.7)	(0.59–87)	(0.01–96.7)	(0.59–87)
	Median	3.7	3.2	17.9	3.1	15.1
	Mann–Whitney test value		9848.500		11,078.000	
	*P* value		0.000		0.000	

*SD* Standard Deviation, *ASA* American Society of Anesthesiology, *NELA* National Emergency Laparotomy Audit, *P-POSSUM* Portsmouth-Physiological and Operative Severity Score for the enumeration of Mortality and Morbidity

### Postoperative mortality

For 90-day mortality, the estimated observed risk was 13.13% (88/670) while for 30-day mortality it was 10.3% (69/670). The predicted mortality hazard was determined using the preoperative NELA score, preoperative P-POSSUM score, and postoperative NELA score. The results were 3.2% (0.01–84.9), 3.7% (0.01–96.7), and 4.8% (0.01–98.7), respectively. The O/E ratio for 30-day mortality was measured as 3.25 for preoperative NELA, 2.81 for preoperative P-POSSUM, and 2.17 for postoperative NELA. The O/E ratio for 90-day mortality was determined to be 4.43 for preoperative NELA, 3.84 for preoperative P-POSSUM, and 2.96 for postoperative NELA. The aforementioned results demonstrated that all models (Table 2) underestimated the likelihood of mortality between 30 and 90 days.

**Table 2:** Ratio of observed deaths to expected deaths, as well as the area under the curve (AUC), for each of the three techniques used to assess 30-day and 90-day mortality

	**Observed deaths**	**Expected deaths**	**O/E ratio**	**AUC**	**Std. error**	***P* value**	**95% CI**	
							Lower bound	Upper bound
**30-day mortality**								
**Preoperative NELA**	10	3.2	3.25	0.774	0.028	*p* < 0.001	0.719	0.829
**Preoperative P-Possum**	10	3.7	2.81	0.763	0.022	*p* < 0.001	0.719	0.806
**Postoperative NELA**	10	4.8	2.17	0.780	0.025	*p* < 0.001	0.730	0.829
**90-day mortality**								
**Preoperative NELA**	14	3.2	4.43	0.649	0.035	*p* < 0.001	0.581	0.717
**Preoperative P-Possum**	14	3.7	3.84	0.782	0.023	*p* < 0.001	0.737	0.828
**Postoperative NELA**	14	4.8	2.96	0.663	0.028	*p* < 0.001	0.608	0.718

*AUC* Area under curve, *Std. Error* Standard error, *NELA* National Emergency Laparotomy Audit, *P-POSSUM* Portsmouth-Physiological and Operative Severity Score for the enumeration of Mortality and Morbidity

### Comparisons between models

#### 30-day mortality

**Fig. 1:**
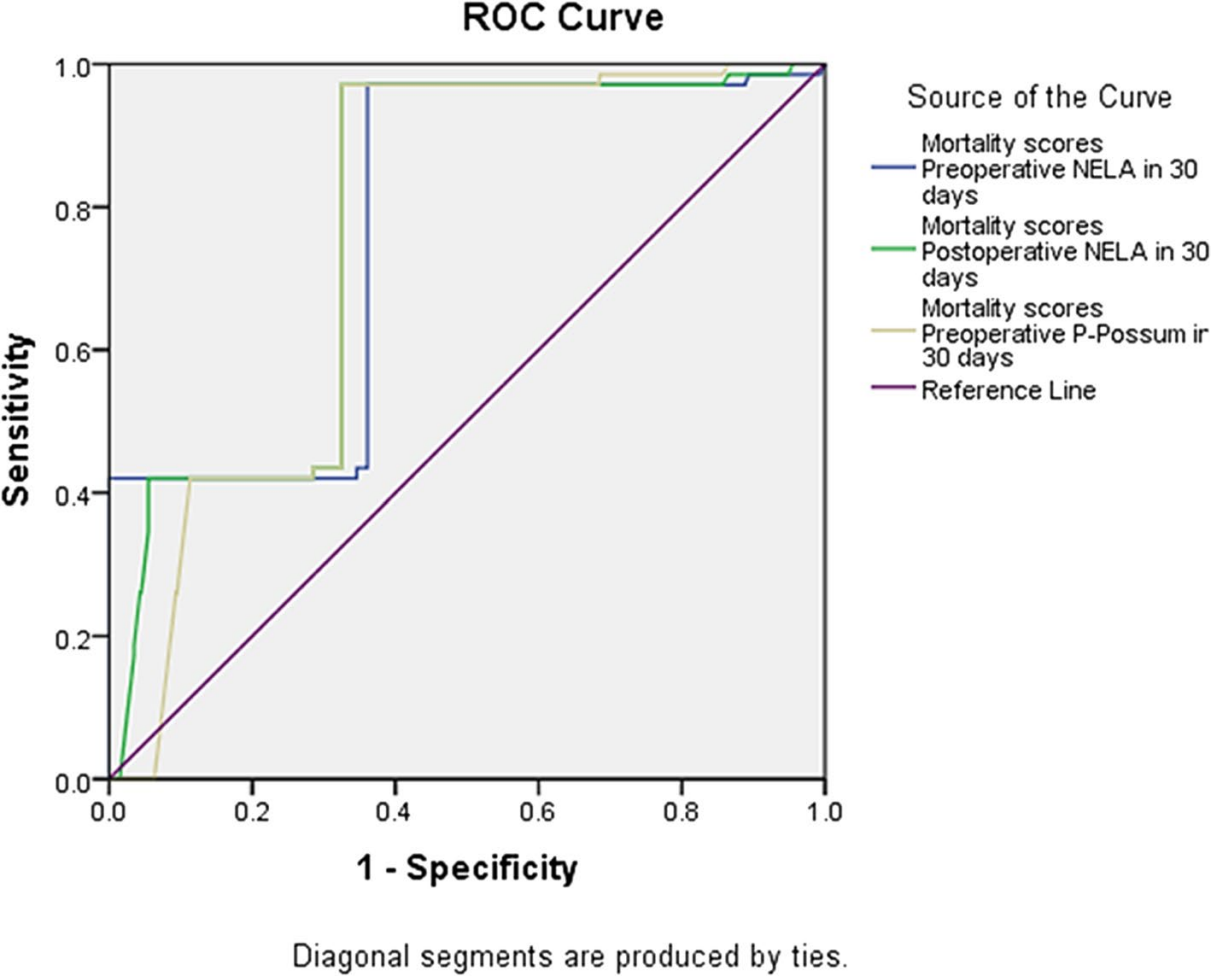
ROC curve of the 3 models in contrast to noticed 30-day mortality

The AUC for the preoperative NELA score was 0.774 (95% confidence interval (CI) = 0.719–0.829, p 0.001), the preoperative P-POSSUM score was 0.763 (95% CI = 0.719–0.806, p 0.001) and the postoperative NELA score was 0.780 (95% CI = 0.730–0.829, p 0.001) (Fig. 1). There was statistically substantial variance among the preoperative NELA score and preoperative P-POSSUM score, preoperative NELA score and postoperative NELA score, and preoperative P-POSSUM score and postoperative NELA score (*p* = 0.027, 0.002, and 0.002, respectively). As regard to the Diagnostic Odds Ratio by binary logistic regression for mortality scores, the postoperative NELA at 30 days was risky which each unit increase of postoperative NELA score, the risk of mortality occurred by 2.328 times (Table 3).

**Table 3:** A contrast of the three AUCs and Odds Ratio for the mortality scores against the observed 30-day and 90-day death rates using pairwise comparisons

	**B**	**S.E**	**Wald**	**df**	***P* value**	**Exp(B)**	**95.0% C.I. for EXP(B)**	
**Mortality in 30 days**							**Lower**	**Upper**
**Preoperative NELA**	-0.036-	0.016	4.869	1	0.027	0.965	0.935	0.996
**Postoperative NELA**	0.845	0.275	9.418	1	0.002	2.328	1.357	3.994
**Preoperative P-POSSUM**	-0.886-	0.284	9.746	1	0.002	0.412	0.236	0.719
**Constant**	-2.147-	0.178	146.236	1	0.000	0.117		
**Mortality in 90 days**							**95.0% C.I. for EXP(B)**	
							**Lower**	**Upper**
**Preoperative NELA**	-0.010-	0.007	2.153	1	0.142	0.990	0.977	1.003
**Postoperative NELA**	0.028	0.005	35.278	1	0.000	1.029	1.019	1.038
**Preoperative P-POSSUM**	0.027	0.006	21.716	1	0.000	1.028	1.016	1.040
**Constant**	-4.091-	0.394	108.030	1	0.000	0.017		

*B* Log Odds (Loading Coefficient), *df* (Degree of freedom), *Exp(b)* Odds Ratio,* Std. Error* Standard error, *NELA* National Emergency Laparotomy Audit, *P-POSSUM* Portsmouth-Physiological and Operative Severity Score for the enumeration of Mortality and Morbidity

#### 90-day mortality

**Fig. 2:**
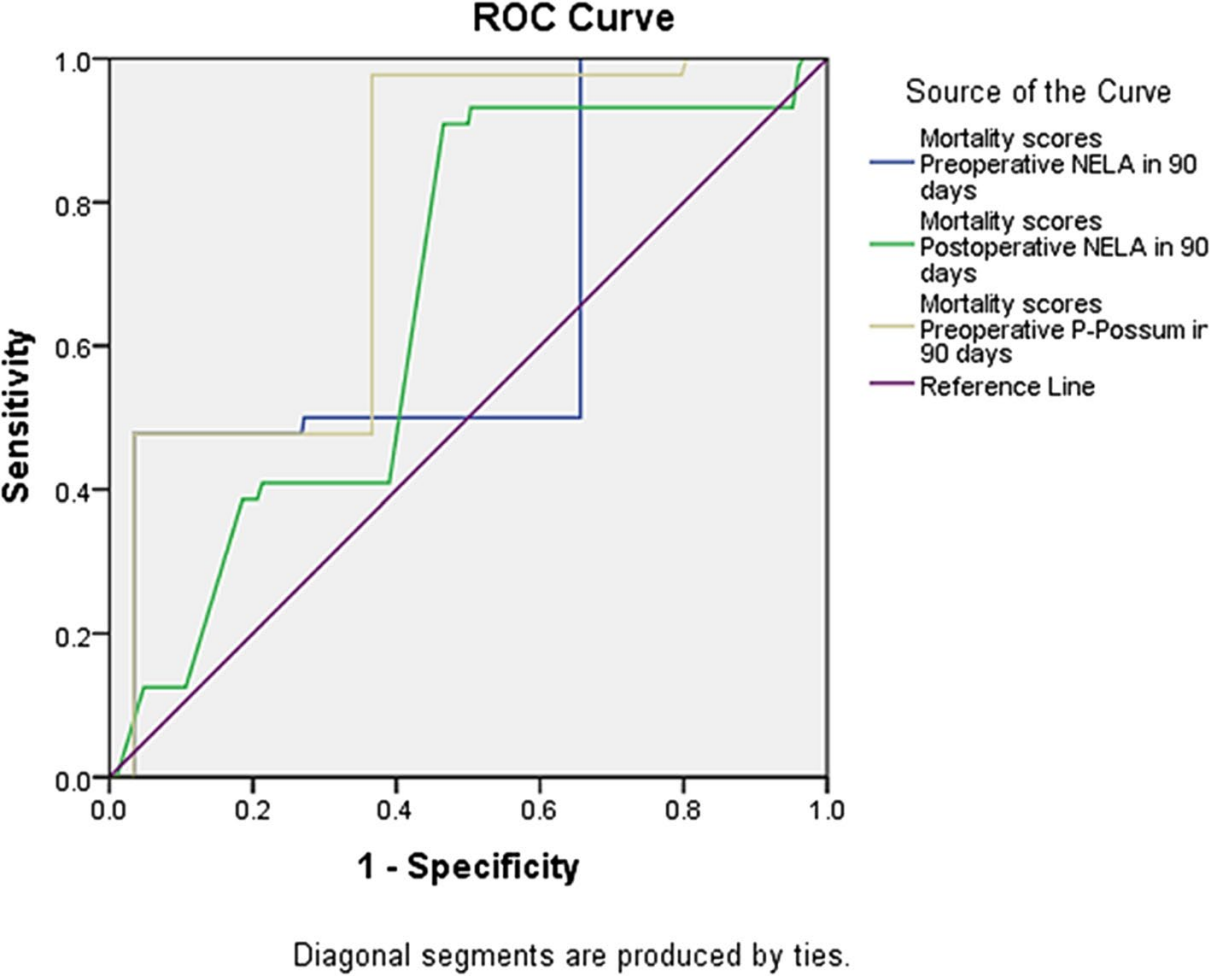
ROC curve of the 3 models in contrast to the noticed 90-day mortality

The preoperative P-POSSUM score was 0.782 (95% CI = 0.737–0.828, p 0.001), the preoperative NELA score was 0.649 (95% CI = 0.581–0.717, *p* < 0.001) and the postoperative NELA score was 0.663 (95% CI = 0.608–0.718, p 0.001), according to ROC curve analysis (Fig. 2). There was no statistically substantial distinction between the preoperative NELA score and preoperative P-POSSUM score (*p* = 0.142) but there was statistical significant difference between preoperative NELA score and postoperative NELA score, and preoperative P-POSSUM score and postoperative NELA score (*p* = 0.001) (Table 3).

All mortality scores at 90 days was more diagnostic than mortality scores at 30 days as the value of -2 Log likelihood at 90 days was less than at 30 days which indicated less error. Moreover, Nagelkerke R Square at 90 days was higher (0.478) which means that the 47.8% of mortality belong to mortality scores parameters at 90 days. The probability of mortality at 90 days due to mortality scores parameters was higher than at 30 days (14.669% and 4.128%, respectively) (Table 4).

**Table 4:** Differences between the probability of mortality at 30-days and 90-days

Variables	Step	-2 Log likelihood	Probability	Nagelkerke R Square
Mortality in 30 days	1	428.990a	4.128%	0.238
Mortality in 90 days	1	267.838a	14.669%	0.478

Probability at 30 days = 4.128% $\frac{Exp (-2.147-0.886 X\left(24.0355\right)+0.845X\left(25.0441\right)-0.036X(24.0261)}{1+Exp (-2.147-0.886 X\left(24.0355\right)+0.845X\left(25.0441\right)-0.036X(24.0261)}$

Probability at 90 days = 14.669% $\frac{Exp (-4.091+0.027 X\left(39.9058\right)+0.028X\left(47.5941\right)-0.010X(24.3277)}{1+Exp (-4.091+0.027 X\left(39.9058\right)+0.028X\left(47.5941\right)-0.010X(24.3277)}$

**Fig. 3:**
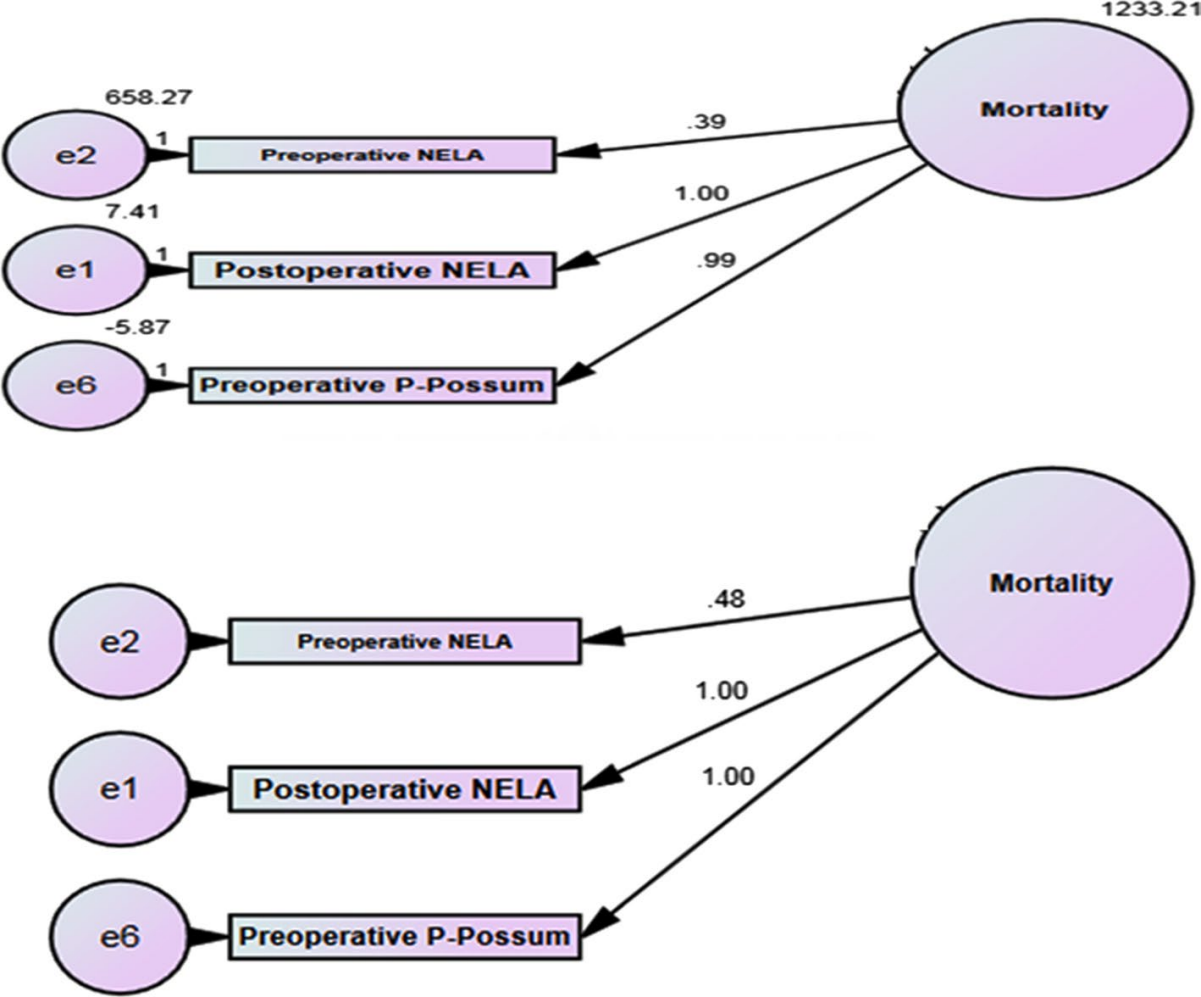
Bootstrap at 30-days mortality

**Fig. 4:**
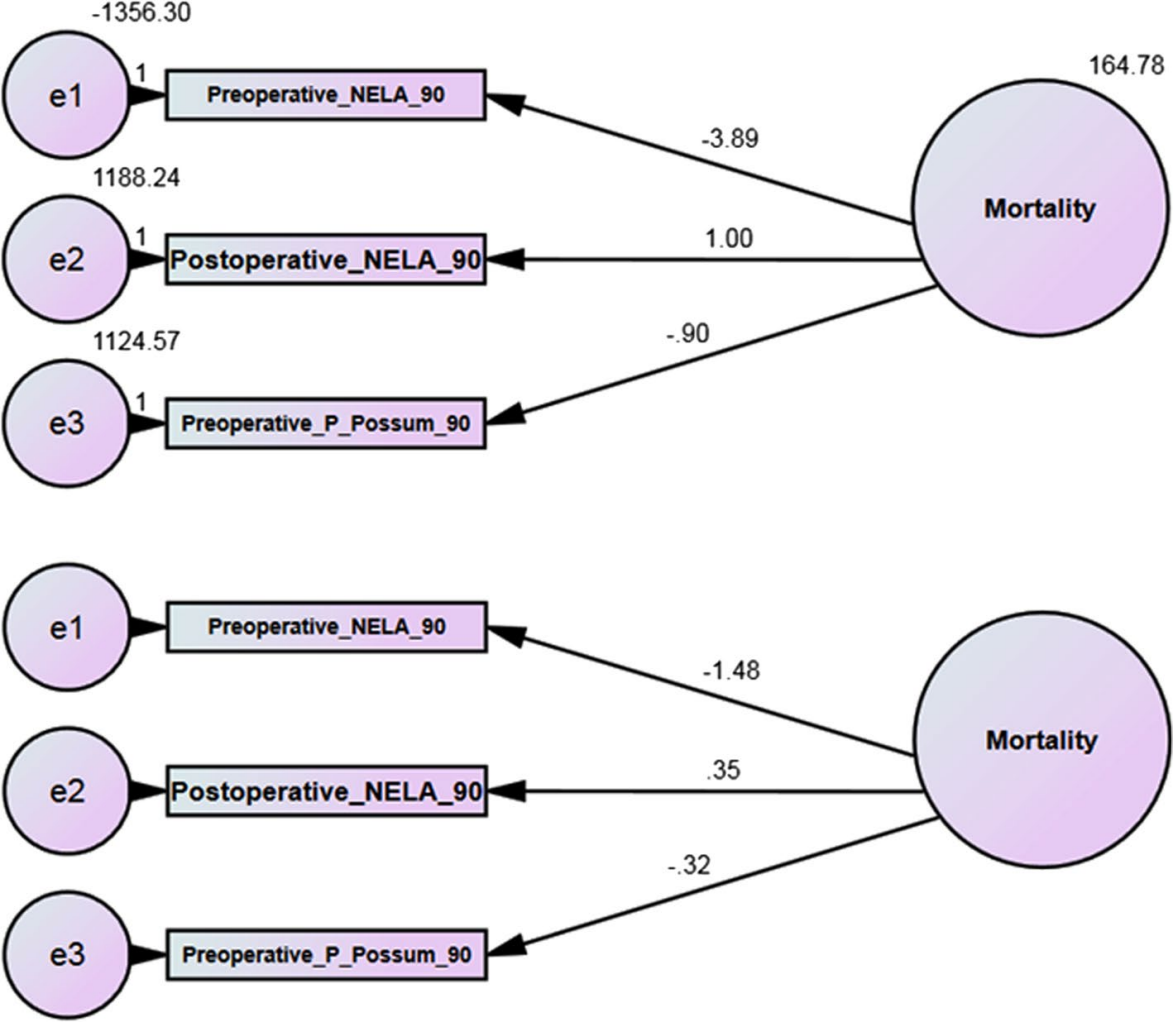
Bootstrap at 90-days mortality

By using the bootstrap through AMOS SPSS program, the value of variation at 90 days was less than 30 days (164.78 and 1233.21, respectively), so the estimation of mortality at 90 days was more accurate than 30 days (Figs. 3 and 4).

## Discussion

Risk assessment plays a significant role in identifying surgical patients at high risk and in setting treatment standards that are appropriate for those risks. One way of identifying high-risk patients is mortality estimates based on risk models such as the NELA and P-POSSUM scores, which may be utilized to determine patients at increased risk and induce their enrollment in hospitals with superior levels of care. We underestimated the risks of 30-day mortality and 90-day mortality in the current study by comparing the effectiveness of the NELA mortality hazard score and the P-POSSUM model in cases experiencing emergency laparotomy. The ability of before-surgery NELA, before-surgery P-POSSUM, and after-surgery NELA scores to predict death after 30 days and death after 90 days in the 670 participants differed statistically significantly, according to pairwise comparisons. Numerous studies [13, 14] have contrasted the PPOSSUM model's effectiveness with that of the NELA score. Thahir et al. evaluated the efficacy of the two models in 650 cases experiencing emergency laparotomies and found that the NELA model had a greater discriminating ability than the P-POSSUM model in forecasting 30-day mortality (AUC = 0.82 vs. 0.77). Additionally, they asserted that the NELA mortality score underestimated the hazard whereas the P-POSSUM overestimated it [13]. In a different study, Lai et al. contrasted the two scores' capacity to accurately predict outcomes in 830 cases and found no significant difference in the discriminative power of the NELA score and P-POSSUM model (AUC = 0.86 vs. 0.84) and they demonstrated that both models significantly underestimated the risks of 30-day mortality [14]. In the preceding studies, the accuracy of the models' projections for 90-day mortality was not evaluated. This may be because the initial intent of the NELA mortality score was to assess the likelihood of dying within 30 days of an emergency abdominal operation.

Using comparison data from the current study and prior research, it can be concluded that the P-POSSUM model and the NELA model are equally good at forecasting outcomes. Lai et al. recommended that the NELA score be used instead of the P-POSSUM model since death rates were inflated and associated with the NELA score were lower than those associated with the PPOSSUM model [14]. Both our data and that of Thahir et al. [13] show that the NELA model underestimated the likelihood of mortality in both trials, therefore this argument cannot stand. Since the NELA mortality score still underestimates mortality rates at 30 and 90 days after laparotomy, it must be enhanced. After surgery 30-day and 90-day mortality was predicted utilizing both the NELA mortality score and the Hajibandeh Index (HI), which is based on the sum of C reactive protein, neutrophils, lactate, lymphocytes, and albumin levels. Patients above the age of 80 who required emergency laparotomy had a better prognosis using the HI than the NELA score, corresponding to research by Hajibandeh et al. [15]. The two scores had similar performances in other respects. Although both the HI and NELA models provide similar results, the authors of the latter argue that this suggests that the NELA model is lacking in some of the necessary parameters [15]. These findings could imply that present mortality prediction tools fail to sufficiently account for modern mortality predictors and that future research should focus on developing and validating scoring systems that account for all modern mortality predictors in cases having emergency laparotomies, including age over 80, sarcopenia, HI, a requirement for bowel resection, an ASA status over 3 and the existence of intra-peritoneal contamination. [16–19]. Moreover, these scores didn`t include the postoperative complications as wound infection, respiratory or cardiac issues and other adverse events so, it is important to focus on developing other scoring systems to estimate the effect of these parameters on mortality.

There are some limitations in our study such as it might not be the best representative of the entire population because it was a single-center study. Moreover, we excluded 54 patients because they did not have all the required investigations to calculate the NELA or P-POSSUM scores; this should be taken into account when interpreting our findings. On the other hand, the sample size was relatively large enough to determine the accuracy of the performance of the NELA and P-PPOSSUM mortality models. Also, the consideration of 90-day mortality in addition to 30-day mortality and evaluation of the performance of postoperative NELA score beside the performance of preoperative NELA and PPOSSUM scores were the strengths of this study.

## Conclusions

In the current study, we demonstrated that there were appreciable variances between the 30-day and 90-day mortality predictions given by preoperative NELA, preoperative P-POSSUM, and postoperative NELA scores. The likelihood of death between 30 and 90 days was underestimated by all models. Patients undergoing emergency laparotomy have a high mortality rate, thus future research should focus on developing and verifying scoring systems that account for all of these parameters.

## Data Availability

The datasets used and/or analyzed during the current study are available from the corresponding author upon reasonable request.
